# The alternative polyadenylation factor CFIm25 orchestrates macrophage antibacterial activity against *Salmonella* through the TAB2 and TBL1XR1 regulators of MAPK and NF-κB signaling

**DOI:** 10.1128/iai.00361-26

**Published:** 2026-07-22

**Authors:** Atish Barua, Srimoyee Mukherjee, Jeff Bourgeois, Claire L. Moore

**Affiliations:** 1 Department of Developmental, Molecular, and Chemical Biology, Tufts University School of Medicine 12261, Boston, Massachusetts, USA; 2 Department of Biology and Biotechnology, Worcester Polytechnic Institute 8718, Worcester, Massachusetts, USA; University of California San Diego School of Medicine, La Jolla, California, USA

**Keywords:** *Salmonella*, macrophage, NUDT21, TAB2, CFIm25, alternative polyadenylation

## Abstract

Macrophage antimicrobial programs are regulated not only by transcriptional networks but also by RNA processing mechanisms affecting signal transduction and effector responses. One such mechanism, alternative polyadenylation (APA), determines mRNA fate by changing the length of the 3′ untranslated region (3′ UTR). However, our understanding of the impact of APA on antibacterial functions and how we can manipulate it to influence infection outcomes remains limited. In this study, we identify the APA regulator CFIm25 (NUDT21) as a promoter of macrophage defense against *Salmonella enterica* serovar Typhimurium (STM). STM infection drives macrophages toward an M2-like immunosuppressive state conducive to bacterial survival, and we show that CFIm25 levels are concurrently reduced during this transition. Overexpression of CFIm25 in infected macrophages blocks STM-induced 3′ UTR lengthening of the key immune regulators TAB2 and TBL1XR1, restoring their mRNA and protein expression. Sustained CFIm25 activity reduces intracellular bacterial burden, enhances macrophage survival, increases reactive oxygen species and nitric oxide production, suppresses arginase activity, and preserves M1-associated surface marker expression and pro-inflammatory cytokine secretion. Mechanistically, TAB2 and TBL1XR1 knockdown studies demonstrate that the antibacterial effects of CFIm25 depend on these CFIm25 targets, which are important for activating MAPK and NF-κB signaling pathways. Furthermore, CFIm25 depletion increased the recovery of intracellular STM from the infected cells. Together, these findings identify APA regulation as a potential target for boosting innate immune defenses against chronic bacterial infections.

## INTRODUCTION

Macrophages play a vital role in the innate immune system by coordinating host antibacterial defense through processes like phagocytosis, production of reactive oxygen and nitrogen species, and release of inflammatory mediators (1). Depending on the signals they receive, macrophages mature in a process called polarization into distinct functional phenotypes ranging along a continuum from a pro-inflammatory (often referred to as M1 or classically activated) state to an anti-inflammatory (M2 or alternatively activated) state (2, 3). M1 macrophages combat microbes with strong antimicrobial and inflammatory responses, characterized by high levels of inducible nitric oxide synthase, reactive oxygen species (ROS), TNF-α, and IL-12 (2). In contrast, M2 macrophages support tissue repair and help resolve inflammation, expressing markers such as arginase, CD206, IL-10, and TGF-β. Balancing macrophage polarization is critical in eliminating pathogens while preventing immune-mediated tissue damage (3).

Phagocytosed bacteria, such as *Salmonella enterica* serovar Typhimurium (STM), have evolved strategies to manipulate macrophage behavior, thereby creating an environment that supports their survival (4–6). In some of the infected cells, the internalized bacteria reprogram the macrophages to suppress M1 bactericidal responses as well as push the macrophages toward an M2-like state that is permissive for STM survival and replication. This shift requires activation of the *Salmonella* pathogenicity island 2 (SPI-2) type 3 secretion system to deliver various effector proteins into the host cell cytoplasm. These infected M2 macrophages play a key role in disease progression because they enable a reservoir of persister bacteria and promote the spread of the bacteria to distant sites, leading to systemic infection (4–7). However, other macrophages manage to lyse internalized bacteria before successful SPI-2 deployment or undergo programmed cell death after bacterial entry (8). In addition, some macrophages are exposed to bacteria or bacterial products but are not infected. These bystander cells can enter an inflammatory M1 polarization state primed for bacterial elimination.

The STM effectors and macrophage signaling pathways that contribute to these different macrophage responses have been well studied (4–6, 8, 9). For example, several effectors inhibit the NF-κB signaling needed for the inflammatory response, and the SteE/SarA protein redirects the action of the GSK3 kinase to activate the transcription regulator STAT3, which in turn promotes bacteria-favorable M2 properties. While there are reports on transcriptional regulation, pathogen control of host mRNA synthesis steps beyond transcription initiation remains poorly understood. Recent studies have highlighted alternative polyadenylation (APA) as an underappreciated regulatory layer influencing immune cell phenotypes at the level of mRNA processing (10). APA produces mRNA isoforms with 3′ untranslated regions (3′ UTRs) of different lengths, which alter the inclusion of sequence elements that affect mRNA stability, translation, and localization (11, 12).

Previously, we have found that the protein CFIm25 (NUDT21 or CPSF5), which governs 3′ UTR length by altering the poly(A) site position, is a key regulator of monocyte differentiation and macrophage polarization (13, 14). However, the role of CFIm25 in modulating bacterial infection of macrophages has not been investigated. In the current study, we show that after STM infection, the CFIm25 level is reduced, and the 3′ UTRs of CFIm25 targets encoding key immune proteins, such as TAB2 and TBL1XR1, are lengthened, suggesting a role for APA changes in the response to STM. Furthermore, restoring CFIm25 levels promotes macrophage survival and antibacterial activity during STM infection. These CFIm25-mediated effects are dependent on TAB2 and TBL1XR1. Our findings identify APA regulation by CFIm25 as a potent mechanism that sets the macrophages up to better destroy incoming bacteria and to resist the *Salmonella* push to an M2-like state. This study also highlights that APA is a crucial, modifiable immune regulatory axis, opening new strategies to boost macrophage antibacterial responses against persistent intracellular infections.

## MATERIALS AND METHODS

### Cell culture and differentiation

THP-1 cells (ATCC TIB-202) were cultured in RPMI-1640 medium with 10% heat-inactivated fetal bovine serum, 2 mM L-glutamine, and 1% penicillin-streptomycin at 37°C in a humidified chamber with 5% CO_2_. To induce differentiation into naive (M0) macrophages, cells were treated with 3 nM phorbol 12-myristate 13-acetate (PMA, Sigma-Aldrich) for 24 h. Fresh media were then added, and after 2 days, polarization toward the M1 phenotype was induced by treating differentiated naive macrophages with 50 ng/mL lipopolysaccharide (LPS) and 10 ng/mL interferon-γ (IFN-γ) for a further 48 h. Polarization toward the M2 phenotype was induced by culturing naive macrophages with 25 ng/mL each of interleukin-4 (IL-4) and interleukin-13 (IL-13) for 48 h. For generation of lentiviruses, HEK293FT cells (Thermo Fisher Scientific, RRID: CVCL_6911) were maintained in high-glucose DMEM supplemented with 10% heat-inactivated fetal bovine serum, 2 mM L-glutamine, and 1% penicillin-streptomycin at 37°C in a humidified incubator with 5% CO_2_.

### Bacterial culture and infection

Preparation of bacteria for infection was based on the protocol described by Jaslow et al. (15). Briefly, *Salmonella enterica* serovar Typhimurium strain SL1344 was cultured overnight in Luria-Bertani (LB) broth at 37°C with shaking and then subcultured at a 1:33 dilution ratio for 3 h. The bacteria were washed with PBS and resuspended in antibiotic-free RPMI-1640 medium. Prior to infection, macrophages were washed and incubated in antibiotic-free RPMI-1640 medium for 1 h at 37°C to allow residual antibiotics to efflux and were maintained in antibiotic-free medium during the infection period. Macrophages were infected at an MOI of 1 for 30 min, washed three times with PBS, and then cultured in a medium containing 100 μg/mL gentamicin for 1 h to remove extracellular bacteria. Afterward, they were maintained in a medium containing 25 μg/mL gentamicin for the remainder of the experiment and harvested at 2 and 6 h post-infection. All time points were calculated from the start of infection, such that the 6-h condition represents a total of 6 h from the initial bacterial exposure.

### Lentivirus construction and transfection

Lentiviral overexpression (OE) and knockdown (KD) of CFIm25 were performed as previously described (14). Briefly, the CFIm25 OE lentiviral vector, kindly provided by Shervin Assassi (University of Texas Health Science Center at Houston, TX, USA), was generated by cloning the coding sequence of human CFIm25 into the pLV-EF1a-IRES-Puro vector (Addgene) (16). For CFIm25 KD, shRNA targeting CFIm25 was cloned into the LT3-GEPIR vector. Control, CFIm25 OE, and CFIm25 KD lentiviruses were generated using the Dharmacon Trans-Lentiviral packaging system in HEK293FT cells and used to transduce monocytic cells in the presence of polybrene. Stable cell lines were selected with puromycin and expanded for subsequent experiments.

### siRNA transfection

For siRNA knockdown experiments, THP-1 cells were differentiated into M0 macrophages as previously described. These M0 cells were then transfected with either gene-specific or non-targeting control siRNAs using jetOPTIMUS transfection reagent (Polyplus), following the manufacturer’s instructions. Briefly, siRNAs were diluted in Opti-MEM and mixed with the transfection reagent for 10–20 min at room temperature, then added to M0 macrophages in antibiotic-free RPMI-1640 medium. Cells were exposed to the siRNA complexes for approximately 6 h, after which the medium was replaced with complete growth medium. Subsequently, the cells were subjected to infection assays as indicated.

### ELISA

The BioLegend Legend Max Kit was used to perform ELISA following the manufacturer’s protocol. In this sandwich ELISA, human TNF-α-, TGF-β-, IL-10-, or IL-12-specific monoclonal antibodies are pre-coated on a 96-well strip plate. Supernatants were collected from cells to measure TNF-α, TGF-β, IL-10, and IL-12 levels, expressed as picograms per milliliter.

### Western blotting

Western blot was performed using total cell extracts from naive and polarized THP-1 cells. Briefly, all cells in the culture dish were collected and then lysed in RIPA buffer (20 mM Tris–HCl, pH 7.5, 150 mM NaCl, 1 mM EDTA, 1 mM EGTA, 1% NP-40, 1% sodium deoxycholate, 2.5 mM sodium pyrophosphate, 1 mM β-glycerophosphate, 1 mM Na_3_VO_4_, and 1 µg/mL leupeptin), and protein concentrations were determined using the BCA assay (Pierce, Thermo Fisher Scientific). Equal amounts of protein (50–80 µg) were separated on 10% SDS-polyacrylamide or Bis-Tris gels and transferred to PVDF membranes. Membranes were blocked for 1 h in 5% non-fat dried milk in Tris-buffered saline with Tween-20 (20 mM Tris–HCl, 150 mM NaCl, pH 7.4, with 0.05% Tween-20) and incubated overnight at 4°C with primary antibodies against CFIm25 (Proteintech, 10322-1-AP), HLA-DR (Santa Cruz, SC 69673), phospho-NF-κB p65 (Ser536) (Cell Signaling, 3033), NF-κB p65 (Cell Signaling, 8242), TAB2 (Cell Signaling, 3744), TBL1XR1 (Novus, NBP1-86996), phospho-STAT1 (Tyr701) (Cell Signaling, 9167), STAT1 (Cell Signaling, 14994), phospho-STAT3 (Tyr705) (Cell Signaling, 9145), STAT3 (Cell Signaling, 4904), IκBα (Cell Signaling, 4814), phospho-p38 (Thr180/Tyr182) (Cell Signaling, 4511), p38 (Cell Signaling, 9212), CD163 (Proteintech, 68218-1-Ig), histone H3 (Cell Signaling, 9715), LL-37 (Santa Cruz, sc-166770), and GAPDH (Santa Cruz, sc-3233). Membranes were then incubated with HRP-conjugated secondary antibodies, developed using enhanced chemiluminescence substrate, and visualized using the LI-COR Odyssey Imaging System. Band intensities were quantified using ImageJ software with GAPDH used as the loading control.

### Flow cytometry

Flow cytometry was performed on PMA-differentiated M0 macrophages with or without infection. All cells in the culture dish were harvested, washed twice with ice-cold FACS buffer (1× phosphate-buffered saline HyClone SH30028 with 2% FBS and 0.5% sodium azide), and first subjected to a standardized gating strategy (Fig. S1). An initial gate based on forward scatter and side scatter (SSC) was used to exclude debris and select the main cell population. From this, singlets were identified using SSC area versus SSC height to remove doublets and aggregates. The resulting single-cell population was used for all downstream analyses, and all reported percentages represent the proportion of events retained relative to the parent population.

For surface marker analysis, cells were stained with fluorochrome-conjugated antibodies CD80 (BioLegend, 305208) and CD206 (BioLegend, 321110). For cell death analysis, cells were fixed in ice-cold 70% ethanol and stored at 4°C for at least 24 h. Fixed cells were washed twice with ice-cold FACS buffer and resuspended in cell cycle staining solution containing RNase A and propidium iodide (Sigma-Aldrich). Samples were incubated in the ice-cold FACS buffer prior to acquisition and analyzed for DNA content within 30 min of PI addition. For ROS detection, cells were incubated with 10 μM 2′,7′-dichlorofluorescin diacetate (Sigma-Aldrich) for 30 min at 37°C and analyzed by flow cytometry. For nitric oxide (NO) detection, cells were incubated with 5 μM 4-amino-5-methylamino-2′,7′-difluorofluorescein diacetate (DAF-FM DA, Invitrogen) for 30 min at 37°C prior to analysis. All data were acquired using a BD LSRII flow cytometer and analyzed using FlowJo software (Tree Star). A minimum of 10,000 events were collected per sample.

To evaluate CFIm25 protein expression following STM infection for Fig. S2D, flow cytometric analysis was performed using the untagged rabbit anti-CFIm25 primary antibody (Proteintech 10322-1-AP) and a secondary PE antirabbit IgG antibody (BioLegend 406421). The cells were scraped, washed twice in PBS, and resuspended in 300–500 µL of ice-cold FACS buffer. They were analyzed on a Cytek Aurora spectral flow cytometer using SpectroFlo software, with acquisition of 10,000–20,000 viable single-cell events per sample, and instrument settings consistent within each experiment. Spectral data were exported to FlowJo (v10.9) for analysis.

### Arginase activity assay

Arginase activity was measured using the Arginase Activity Assay Kit (Sigma-Aldrich), which detects the conversion of arginine into urea and ornithine. The urea produced reacts with the kit’s substrate, creating a colored compound that indicates arginase activity. Cells were lysed in 100 μL of lysis buffer with protease inhibitors. After centrifugation at 10,000 × *g* for 10 min, the supernatant was collected, and arginase activity was determined according to the manufacturer’s instructions. The color change was read at 430 nm and normalized to total protein content.

### Bacterial colony-forming unit assay

Infected macrophages were lysed with 1% Triton X-100 in PBS. Serial dilutions were plated on LB agar and incubated at 37°C overnight. Colonies were counted and expressed as colony-forming units per milliliter.

### Nuclear-cytoplasmic fractionation

Nuclear and cytoplasmic fractions were isolated using the NE-PER Nuclear and Cytoplasmic Extraction Reagent Kit (Thermo Scientific) according to the manufacturer’s instructions.

### RT-qPCR analysis for APA and expression

RNA isolation was performed from THP-1 macrophages as previously described (13). Total RNA was extracted from 2 × 10^6^ cells using TRIzol reagent according to the manufacturer’s instructions. RNA (1.5 µg) underwent reverse transcription with an oligo dT primer and Superscript III reverse transcriptase. The resulting cDNA was amplified via qPCR with specific primers (Table S1) and was quantified using the ΔΔCt method. ACTB RNA served as the normalization control for RT-qPCR-based RNA expression analyses. Primers targeting total or long transcripts were designed using the poly(A) site annotations available in poly(A)_DB that were also confirmed by 3′-end sequencing, as described in detail in our previous work (17).

### Statistical analysis

Experiments were performed with at least three independent sets. Data are presented as mean ± SE. Statistical analysis was conducted using GraphPad Prism 6.01 (GraphPad Software Inc., La Jolla, CA, USA). A two-way ANOVA was used to compare group differences. Significance levels were *P ≤ 0.05, **P ≤ 0.01, ***P ≤ 0.001. A *P* value of less than 0.05 was considered statistically significant.

## RESULTS

### *Salmonella* Typhimurium infection downregulates CFIm25 as it shifts macrophages to a replication-permissive state

**Fig 1:**
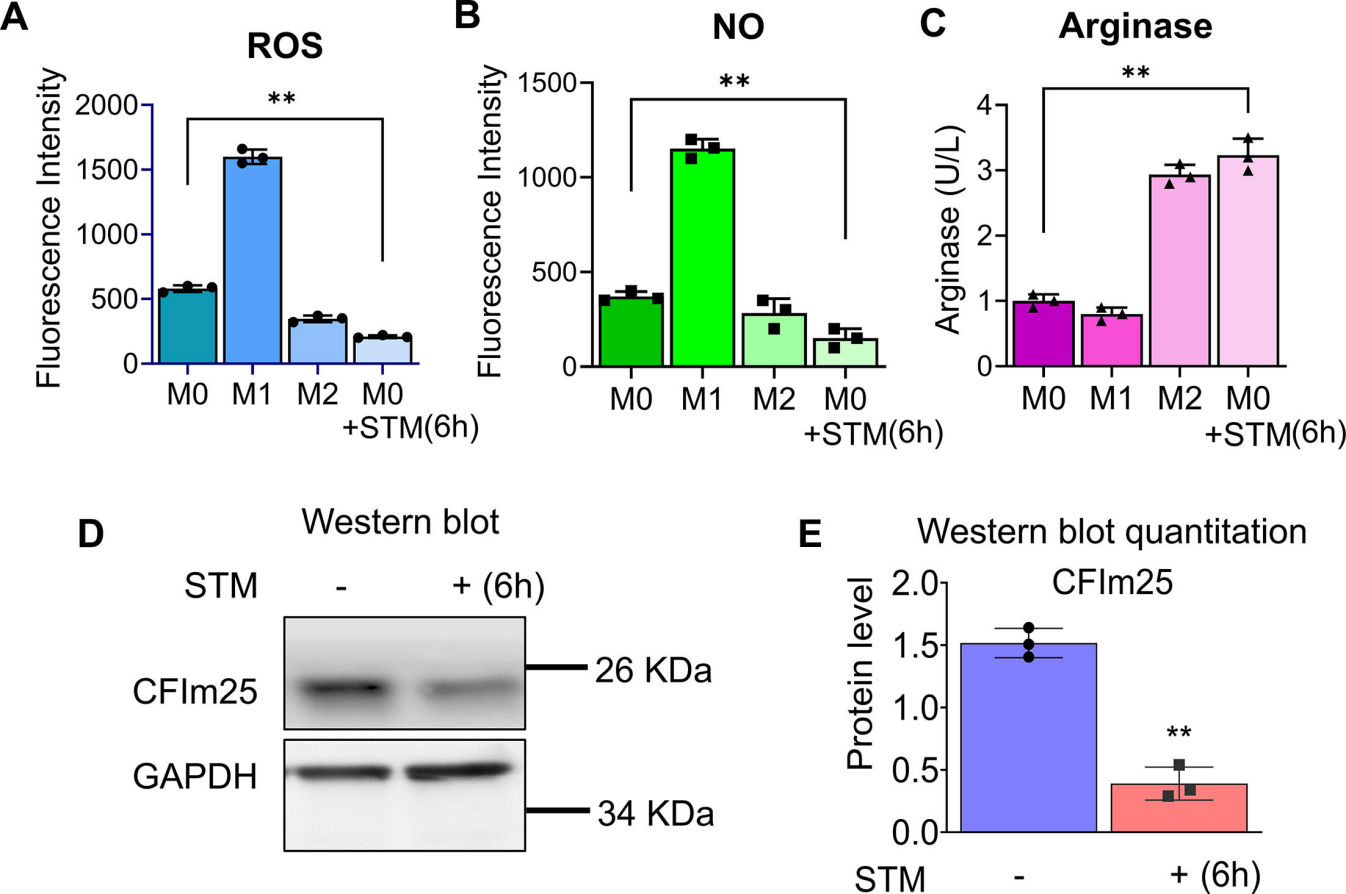
*Salmonella* Typhimurium infection for 6 h suppresses CFIm25 expression and alters macrophage polarization-associated functional responses. (**A**) Flow cytometric quantification of reactive oxygen species (ROS) production using the 2′,7′-dichlorofluorescin diacetate fluorescence assay in uninfected M0, classically polarized M1 (LPS + IFN-γ), alternatively polarized M2 (IL-4 + IL-13), and *Salmonella* Typhimurium-infected macrophages (M0 + STM). (**B**) Flow cytometric quantification of nitric oxide (NO) production using the DAF-FM DA fluorescence assay in M0, M1, M2, and M0 + STM macrophages. (**C**) Arginase activity measured by urea production in M0, M1, M2, and M0 + STM macrophages. (**D**) Western blot analysis of CFIm25 protein expression in THP-1 macrophages 6 h after STM infection compared to uninfected controls. A representative immunoblot is shown, with molecular weight markers indicated. (**E**) Densitometric quantification of CFIm25 protein levels normalized to GAPDH. Data are presented as means ± SD from three independent biological replicates (*n* = 3). **, *P* < 0.01.

A characteristic of STM infection is its ability to push macrophage populations toward an M2-like, replication-permissive state (5). This transition to an M2 state is reminiscent of the changes we had observed in macrophages when CFIm25 was depleted (14). To determine if the CFIm25 level was altered by bacterial infection, PMA-differentiated THP-1 macrophages (M0) were infected with STM at an MOI of 1. To confirm that these macrophages are in the non-activated M0 state, we compared their ROS, NO, and arginase profiles with those of classically polarized M1 (LPS + IFN-γ) and alternatively polarized M2 (IL-4 + IL-13) macrophages (Fig. 1A through C). M0 macrophages displayed ROS and NO levels clearly lower than those of fully polarized M1 macrophages and similar in levels to those seen in the M2 macrophages. Following infection with STM for 6 h (M0 + STM), the macrophage population shifted to more closely resemble M2 polarization: elevated arginase and low ROS/NOS. Western blotting showed significant depletion of CFIm25 protein within 6 h post-infection (Fig. 1D and E). These findings are consistent with a model in which STM actively suppresses CFIm25 as part of its strategy to impair macrophage antimicrobial functions.

### CFIm25 overexpression blocks the STM-driven shift to permissive M2 macrophages and maintains antibacterial activity during infection

**Fig 2:**
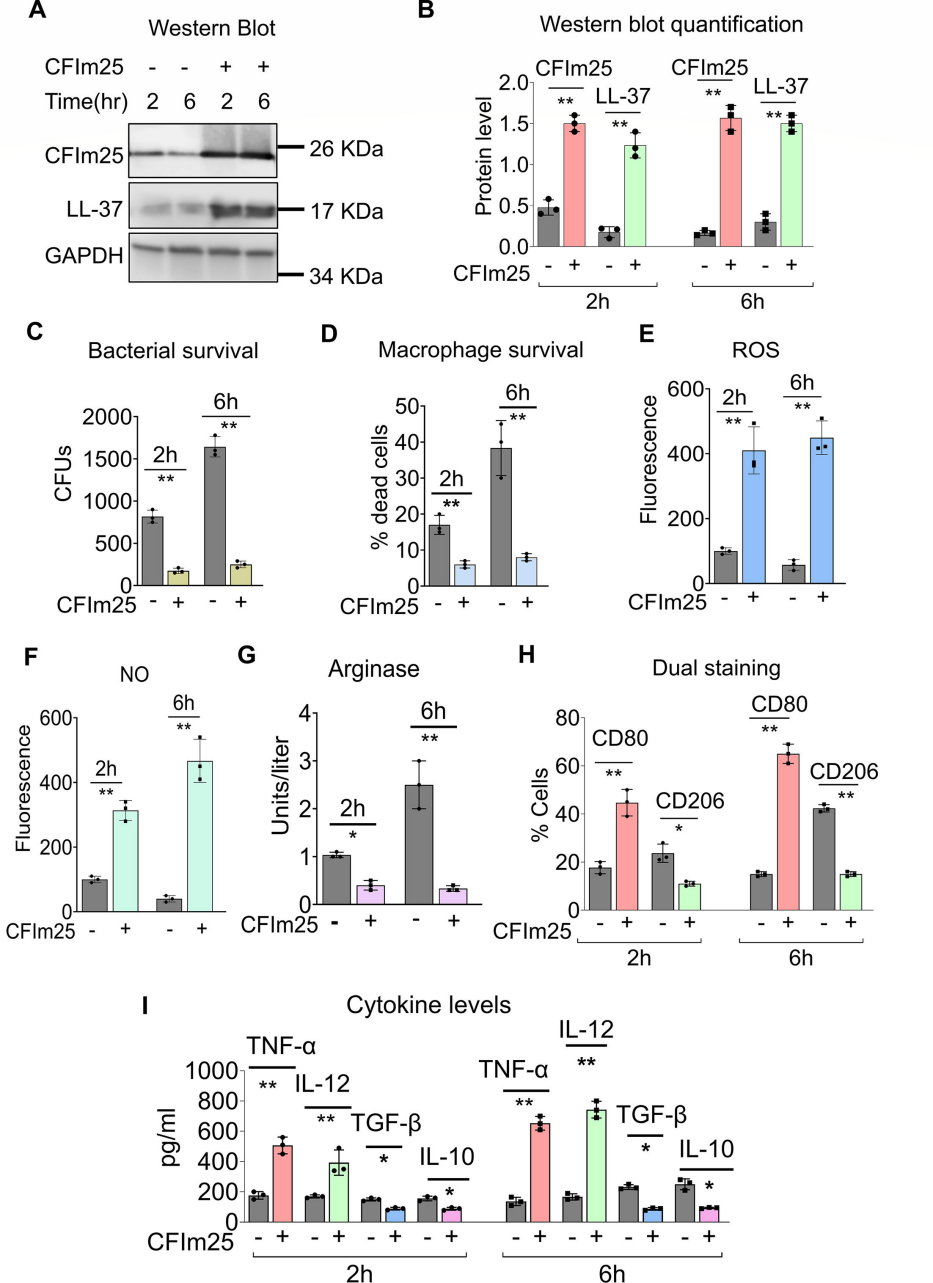
CFIm25 overexpression modulates macrophage antibacterial and polarization-associated responses during *Salmonella* Typhimurium infection. (**A**) Western blot analysis of CFIm25 expression in control (−) and CFIm25-overexpressing (+) THP-1 macrophages infected with STM for 2 and 6 h. Representative immunoblots for CFIm25, LL-37, and GAPDH are shown, with molecular markers indicated. (**B**) Densitometric quantification of CFIm25 and LL-37 protein levels normalized to GAPDH at 2 and 6 h post-infection. (**C**) Intracellular bacterial burden determined by colony-forming unit (CFU) assay in control and CFIm25-OE macrophages at 2 and 6 h post-infection. (**D**) Propidium iodide-based flow cytometric analysis of cell death in control and CFIm25-OE macrophages at 2 and 6 h post-infection. (**E**) Quantification of reactive oxygen species (ROS) production using the 2′,7′-dichlorofluorescin diacetate fluorescence assay in control and CFIm25-OE macrophages at 2 and 6 h post-infection. (**F**) Quantification of nitric oxide (NO) production using the DAF-FM DA fluorescence assay in control and CFIm25-OE macrophages at 2 and 6 h post-infection. (**G**) Arginase activity measured by urea production in control and CFIm25-OE macrophages at 2 and 6 h post-infection. (**H**) Flow cytometric analysis of M1 (CD80) and M2 (CD206) surface marker expression in control and CFIm25-OE macrophages at 2 and 6 h post-infection. (**I**) ELISA quantification of TNF-α, IL-12, TGF-β, and IL-10 concentrations in culture supernatants from control and CFIm25-OE macrophages at 2 and 6 h post-infection. Data are presented as means ± SD from three independent biological replicates (*n* = 3). *, *P* < 0.05; **, *P* < 0.01.

To test whether CFIm25 can directly counteract the STM-driven shift to permissive M2 macrophages, THP-1 macrophages overexpressing CFIm25 (CFIm25-OE) or control cells without CFIm25-OE were generated by lentiviral transduction and challenged with STM for 2 and 6 h. These time points were selected to assess properties immediately after internalization and during establishment of the protective *Salmonella*-containing vacuole (2 h) and around the start of replication (6 h) (18, 19). Moreover, the strong reduction of CFIm25 at 6 h of infection (Fig. 1D and E) suggested this might be a time when the progress of the infection would be susceptible to altering CFIm25 levels. CFIm25 level was strongly increased in CFIm25 overexpressing cells at 2 h post-infection and remained high at 6 h (Fig. 2A and B).

To determine the consequences of CFIm25 OE on infection outcomes, we assessed how CFIm25 OE affected bacterial survival within the macrophages and the ability of the macrophages to survive the infection. At 6 h, the number of recovered bacteria has approximately doubled compared to 2 h, indicating replication has begun (Fig. 2C). However, CFIm25-OE macrophages demonstrated much greater bacterial clearance at 2 and 6 h post-infection, with approximately 4.7-fold (2 h) and 6.6-fold (6 h) reductions in intracellular bacteria compared with control macrophages (Fig. 2C). We had previously shown that CFIm25 OE had no effect on the viability of M0 macrophages (13). Now, to examine macrophage survival after infection, we determined the percentage of dead cells by their ability to stain with propidium iodide and observed strong resistance to infection-induced cell death in CFIm25-OE macrophages at both 2 and 6 h post-infection (Fig. 2D).

We next examined the consequences of CFIm25 OE on macrophage properties, such as expression of NO and ROS, which are key macrophage defenses against pathogens (1). Consistent with our previous report (14), CFIm25 OE in uninfected cells causes an increase in NO and, as we show here, a similar increase in ROS (Fig. S2A and B). When cells are infected with STM for 2 or 6 h, ROS and NO levels remain low in the absence of CFIm25 OE, but with CFIm25 OE, the levels are much higher at both time points (Fig. 2E and F). When uninfected cells are compared to cells 6 h post-infection, infection causes a decrease in ROS and NO levels in the absence of CFIm25 OE, but with CFIm25 OE, the decline caused by STM is blocked and levels rise even higher upon infection compared to uninfected cells (Fig. S2A and B).

In addition, we measured the level of the antimicrobial peptide LL-37 as another indicator that an antibacterial state has been activated (1), and found that its levels strongly increased in cell lysates when CFIm25 was overexpressed during macrophage infection (Fig. 2A and B). To test CFIm25’s capacity to prevent a transition to a more M2-like, replication-permissive state, we looked at the production of arginase, a defining characteristic of M2 macrophages (3). We found that while arginase was low in uninfected cells (Fig. S2C), controls without CFIm25 overexpression showed an increase in arginase levels after infection. However, the amount of arginase remained low in CFIm25 OE macrophages at both 2 and 6 h post-infection (Fig. 2G; Fig. S2C).

**Fig 3:**
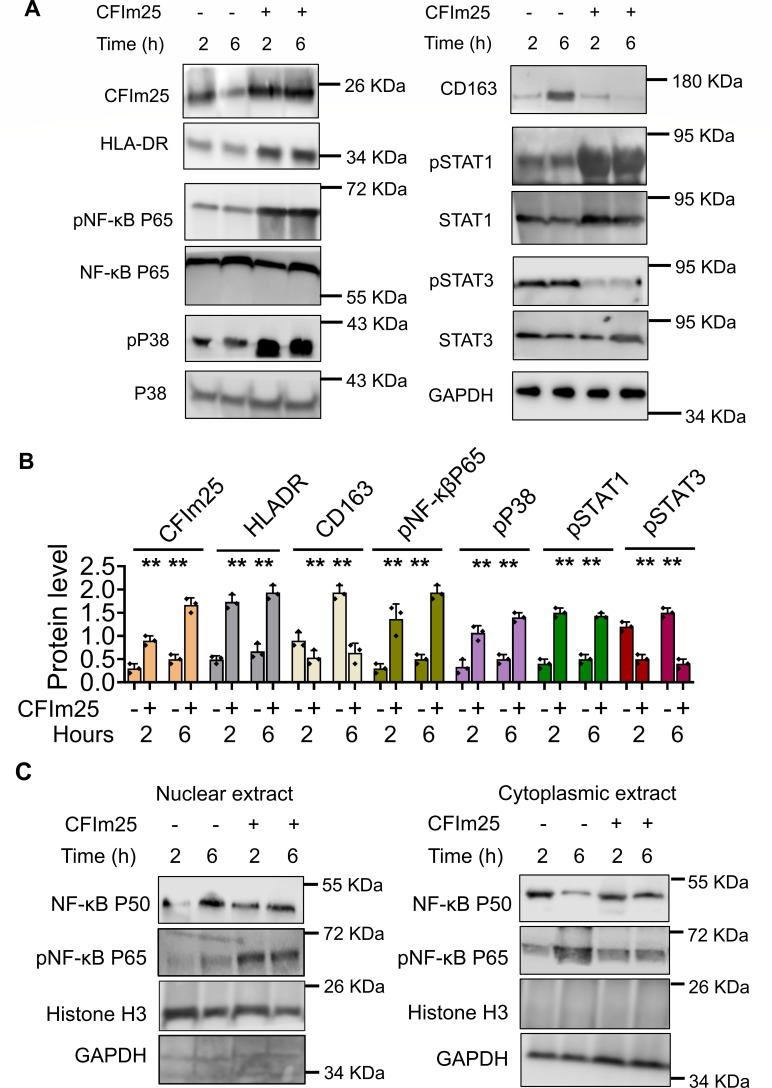
CFIm25 regulates immune signaling pathways during *Salmonella* Typhimurium infection. (**A**) Representative Western blot analysis of CFIm25, HLA-DR, CD163, and the indicated immune signaling proteins and their phosphorylated forms in control (−) and CFIm25-overexpressing (+) macrophages at 2 and 6 h post-infection, with GAPDH used as the loading control. (**B**) Densitometric quantification of protein expression normalized to GAPDH. Data are presented as means ± SD from three independent biological replicates (*n* = 3). **, *P* < 0.01. (**C**) Representative Western blot showing nuclear and cytoplasmic distribution of NF-κB subunits in control and CFIm25-OE macrophages 2 and 6 h post-infection, assessed by fractionation and immunoblotting.

Two additional indicators of polarization are the expression of M1- or M2-specific surface markers and the secretion of characteristic cytokines. Flow cytometric analysis showed that infected CFIm25-OE cells maintained high CD80 (M1) expression and blocked the STM-mediated increase in CD206 (M2) surface expression (Fig. 2H). This shift in polarization state was confirmed by Western blot, which showed that CFIm25 suppressed expression of the CD163 M2 marker while elevating that of the HLA-DR M1 marker (Fig. 3A and B). ELISA indicated sustained secretion of pro-inflammatory cytokines (TNF-α and IL-12) and decreased levels of anti-inflammatory cytokines (IL-10 and TGF-β) during infection of iCFIm25-OE macrophages compared to controls (Fig. 2I). Overall, these findings demonstrate that increasing CFIm25 enhances microbicidal function during bacterial challenge by boosting ROS and NO, promotes macrophage survival, and prevents infection-induced M2 polarization while supporting M1-specific response features.

Our findings also suggest that loss of endogenous CFIm25 may be sufficient to suppress macrophage antimicrobial activity. To test this idea, we used stable CFIm25 knockdown cells and assessed the consequences with and without STM infection. CFIm25 knockdown significantly reduced CFIm25 protein levels both in the presence and absence of infection (Fig. S2D). This depletion also increased the recovery of intracellular STM from the infected cells (Fig. S2E), and supports the idea that bacteria actively exploit the CFIm25-APA axis as an immune evasion strategy.

### CFIm25 affects multiple signaling pathways governing M1/M2 fate

We next examined activation of signaling pathways that regulate M1 and M2 phenotypes by specific kinases. Infected CFIm25 OE macrophages exhibited increased phosphorylation of NF-κB P65, P38 MAPK, and STAT1 (Fig. 3A and B), which drive M1 polarization (20, 21). A hallmark of STM infection is the activation of STAT3, which leads to increased expression of anti-inflammatory genes, such as IL-10 and IL-4Rα, and promotes the M2 state (15, 22, 23). Consistent with CFIm25’s ability to resist the STM-induced push to M2-like cells, CFIm25 OE blocked STAT3 phosphorylation during infection (Fig. 3A and B).

An effective activation of NF-κB-dependent genes requires the translocation of phosphorylated NF-κB P65 into the nucleus, increased association of P65 with the NF-κB P50 subunit, and a decrease in repressive nuclear-localized P50-P50 homodimers (24, 25). To assess the effects of CFIm25 on localization, we conducted nuclear-cytoplasmic fractionation on infected cells with and without CFIm25 overexpression. We confirmed clean fractionation by the absence of GAPDH in the nuclear fraction and absence of histone H3 in the cytoplasmic one (Fig. 3C). This experiment showed that CFIm25-OE macrophages had markedly higher nuclear phosphorylated p65 at 2 and 6 h post-infection compared with controls. In contrast, the control cells showed very little nuclear phosphorylated P65 but increased nuclear NF-κB P50 at 6 h (Fig. 3C).

### TAB2 and TBL1XR1 are critical for CFIm25-mediated antimicrobial defense

**Fig 4:**
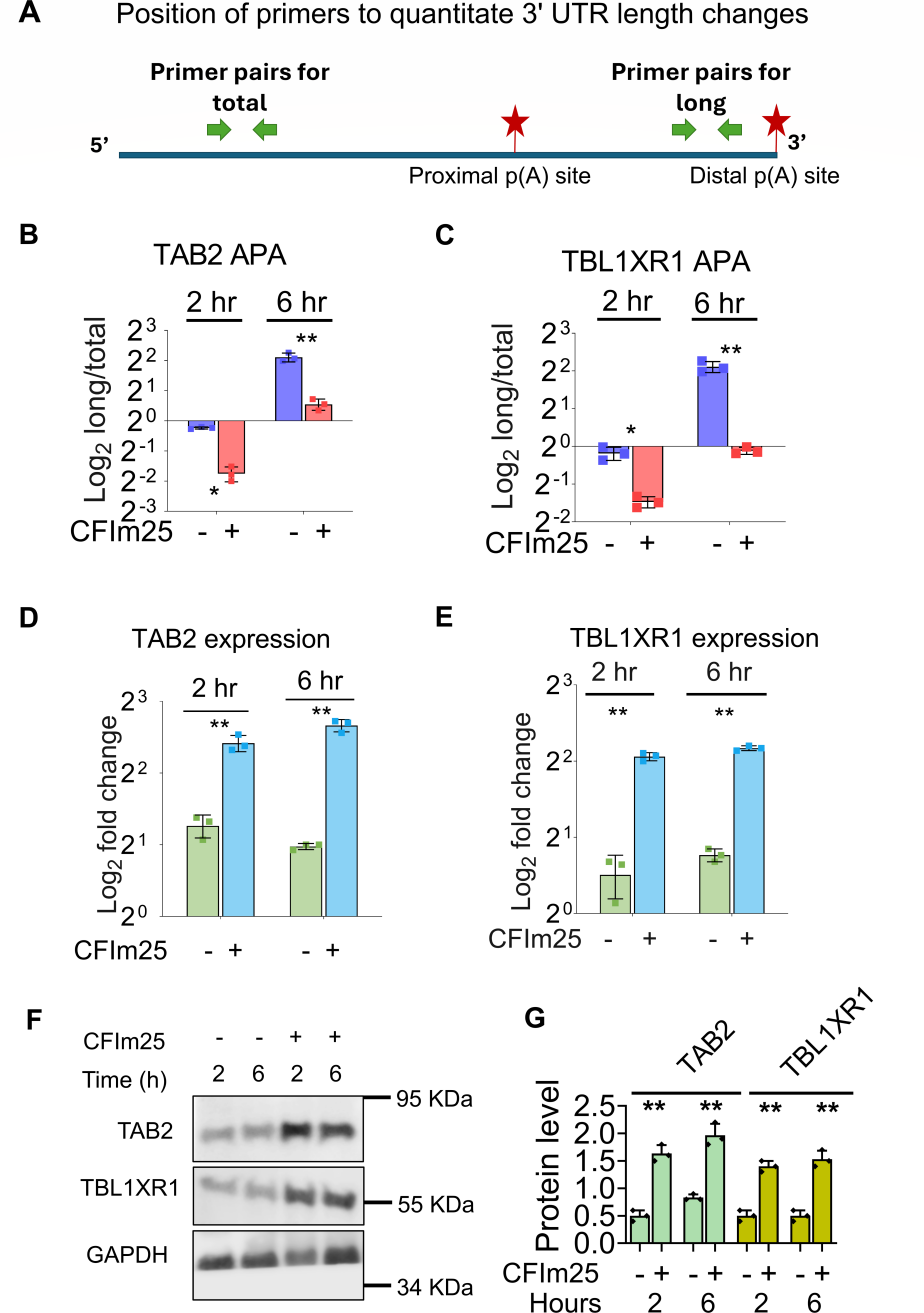
CFIm25 regulates alternative polyadenylation and the expression of key immune regulators. (**A**) Schematic representation of the long/total TAB2 and TBL1XR1 transcript regions targeted for RT-qPCR to assess APA and mRNA expression. The positions of the alternative poly(A) sites, which were determined by 3′-end sequencing and match annotated poly(A) sites (13), are indicated. (**B and C**) RT-qPCR analysis showing the log_2_ ratio of long/total TAB2 and TBL1XR1 mRNA isoforms in control and CFIm25-OE macrophages at 2 and 6 h post-infection, normalized to GAPDH. (**D and E**) TAB2 and TBL1XR1 mRNA expression after STM infection, assessed by RT-qPCR analysis in control (−) and CFIm25-OE (+) macrophages at 2 and 6 h post-infection. Values represent log_2_ fold change normalized to ACTB. (**F**) Western blot analysis of TAB2 and TBL1XR1 in control and CFIm25-OE macrophages at 2 and 6 h post-infection, with GAPDH as the loading control. (**G**) Quantification of changes of the indicated proteins in Western blots of samples from control and CFIm25-OE macrophages at 2 and 6 h post-infection, with values normalized to GAPDH. A representative immunoblot is shown, with molecular weight markers indicated. Data are presented as means ± SD (*n* = 3). *, *P* < 0.05; **, *P* < 0.01.

Our previous study showed that CFIm25 OE in monocytes expedites macrophage differentiation by activating the NF-κB signaling pathway (13). This outcome is associated with shortening of the 3′ UTRs of TAB2 and TBL1XR1 transcripts and increased protein levels of these NF-κB-positive effectors (13). To assess how STM infection influenced APA of these targets, we performed RT-qPCR with primer pairs that detected either total mRNA levels or specifically those with longer 3′ UTRs (Fig. 4A and sequences in Table S1). This analysis revealed that STM infection caused an extension of the 3′ UTRs of TAB2 and TBL1XR1 mRNAs in control cells, consistent with increased use of distal polyadenylation sites (Fig. 4B and C). However, CFIm25-OE cells maintained a lower long/total mRNA ratio during infection, signifying greater use of proximal sites and 3′ UTR shortening (Fig. 4B and C). This shortening was associated with significantly increased transcript (Fig. 4D and E) and protein levels (Fig. 4F and G) of both TAB2 and TBL1XR1.

**Fig 5:**
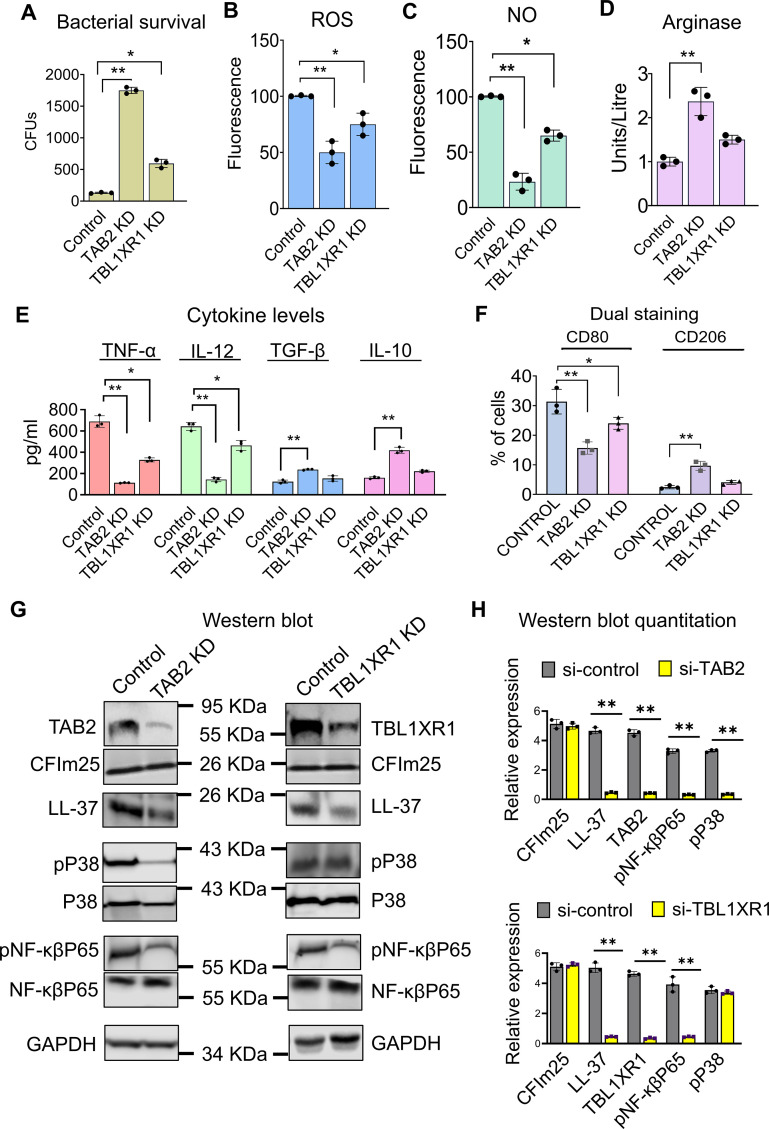
TAB2 and TBL1XR1 are critical for CFIm25-mediated antimicrobial defense. (**A**) Intracellular bacterial burden (CFU) recovered at 6 h post-infection from CFIm25-OE THP-1 macrophages transfected with control siRNA, TAB2 siRNA, or TBL1XR1 siRNA. (**B**) Flow cytometric quantification of ROS production using the 2′,7′-dichlorofluorescin diacetate fluorescence assay in CFIm25-OE macrophages transfected with control, TAB2, or TBL1XR1 siRNA at 6 h post-infection. (**C**) Flow cytometric quantification of NO production using the DAF-FM DA fluorescence assay in CFIm25-OE macrophages transfected with control, TAB2, or TBL1XR1 siRNA at 6 h post-infection. (**D**) Arginase activity measured by urea production in CFIm25-OE macrophages transfected with control, TAB2, or TBL1XR1 siRNA at 6 h post-infection. (**E**) Concentrations of TNF-α, IL-12, TGF-β, and IL-10 in culture supernatants from CFIm25-OE macrophages expressing control, TAB2, or TBL1XR1 siRNAs, harvested at 6 h post-infection and measured by ELISA. (**F**) Flow cytometric analysis of M1 (CD80) and M2 (CD206) surface marker expression at 6 h post-infection in CFIm25-OE macrophages transfected with control, TAB2, or TBL1XR1 siRNA. Graphs show the percentage of cells expressing each marker. (**G**) Western blot analysis of CFIm25, LL-37, pP38, P38, pNF-κB P65, NF-κB P65, and GAPDH in CFIm25-OE macrophages transfected with control or TAB2 siRNA (left) and control or TBL1XR1 siRNA (right) at 6 h post-infection. A representative immunoblot is shown, with molecular weight markers indicated. (**H**) Densitometric quantification of the Western blots normalized to GAPDH. Data are presented as means ± SD from three independent experiments (*n* = 3). *, *P* < 0.05; **, *P* < 0.01.

We previously showed that knockdown of TAB2 and TBL1XR1 in CFIm25-overexpressing cells slowed the differentiation of monocytes to macrophages and attenuated CFIm25-mediated NF-κB activation (13). We now wanted to determine if depletion of TAB2 or TBL1XR1 would block the protective effects of CFIm25 during infection. CFIm25-OE macrophages were transfected with control siRNA or siRNAs against TAB2 or TBL1XR1, followed by STM infection for 6 h and harvesting of cells. TAB2 knockdown produced a striking increase in the amount of recovered bacteria, while TBL1XR1 knockdown caused a more modest increase (Fig. 5A), indicating a reduction in CFIm25-mediated bacterial clearance. TAB2 and TBL1XR1 siRNAs also suppressed ROS and NO levels, with TAB2 siRNA having a stronger effect (Fig. 5B and C). TAB2 knockdown upregulated arginase activity and the anti-inflammatory cytokines TGF-β and IL-10, whereas TBL1XR1 had no or only a modest effect on these M2 properties (Fig. 5D and E). Flow cytometric analysis indicated that TAB2 depletion reduced the M1 marker CD80 and increased expression of the M2 marker CD206, while TBL1XR1 knockdown had a less pronounced effect on surface marker expression (Fig. 5F). TNF-α and IL-12 secretion was suppressed by both knockdowns, with TAB2 siRNA again having a stronger effect (Fig. 5E).

From the Western blot analysis, it is evident that the knockdowns caused a pronounced depletion of TAB2 and TBL1XR1 (Fig. 5G through H). As expected from their known roles in NF-κB signaling, depletions of both proteins reduced the level of phosphorylated P65 (Fig. 5G through H). Consistent with TAB2’s role in activating the P38 MAPK pathway (26, 27), silencing TAB2, but not TBL1XR1, decreased P38 phosphorylation (Fig. 5G through H), indicating a central role for TAB2 in integrating NF-κB and MAPK signaling for antibacterial immunity.

## DISCUSSION

This study highlights the importance of CFIm25, an APA regulator, in determining whether STM-infected macrophages become bactericidal or permit bacterial replication. Upon STM infection, CFIm25 is downregulated at 6 h post-infection, and this change correlates with reduced ROS and NO production and a metabolic shift toward an arginase-rich, M2-like state permissive for bacterial survival. Arginase redirects arginine metabolism away from microbicidal NO production and toward ornithine and polyamines, which enhance the M2 anti-inflammatory responses and support the assembly and function of the SPI-2 type 3 secretion system (28). Conversely, maintaining CFIm25 expression prevents this shift at multiple functional levels. It preserves an M1-like macrophage phenotype evident from the upregulation of M1 markers and the production of ROS, NO, and pro-inflammatory cytokines.

In our experiments, CFIm25 overexpression also elevated the level of the antimicrobial peptide, LL-37, which is produced by activated macrophages and kills microorganisms by membrane disruption (1). *Salmonella* can mount a defense against LL-37 by remodeling its outer membrane, so LL-37 has a harder time getting in and disrupting the cell envelope (29). Thus, in our experiments, the rise in LL-37 may not contribute to the increased bacterial killing observed upon CFIm25 overexpression. Nevertheless, this increase does provide further evidence for the role of CFIm25 in promoting an antibacterial response. Together, our data support a model in which STM actively depletes endogenous CFIm25 to suppress macrophage pro-inflammatory responses and facilitate the permissive M2 state, while sustained CFIm25 activity protects macrophages against STM-induced reprogramming and leads to enhanced intracellular STM elimination and improved macrophage survival.

Mechanistically, the decrease in CFIm25 during infection is associated with lengthening of the 3′ UTRs of mRNAs encoding the critical signaling proteins TAB2 and TBL1XR1. Increasing CFIm25 expression reverses this shift and promotes proximal poly(A) site usage in TAB2 and TBL1XR1 transcripts and an increase in their encoded proteins which regulate NF-κB and P38 signaling. Infected CFIm25 OE macrophages exhibited increased phosphorylation of STAT1, NF-κB P65, and P38 MAPK, as well as increased nuclear localization of pNF-κB P65, consistent with promotion of M1 polarization and the production of antimicrobial agents that suppress intracellular bacterial growth and survival (25, 30–32). In addition, CFIm25-OE blocked STAT3 phosphorylation during infection. STAT3 activation is a hallmark of STM infection that leads to increased expression of anti-inflammatory genes such as IL-10 and IL-4Rα, which promote the M2 state (15, 22, 23). Overall, our findings indicate that CFIm25 maintains a signaling environment that favors M1-like inflammatory polarization.

Large-scale APA data sets now provide a framework for interpreting these mechanistic observations. Quantitative 3′ UTR-APA maps across human immune cell types reveal thousands of immune response-associated 3′ UTR APA quantitative trait loci (3′aQTLs), which link germline genetic variation to cell type-specific shifts in proximal versus distal poly(A) site usage at key immune genes (33, 34). Many of these 3′aQTLs are dynamically unmasked only after stimulation, underscoring that APA is a major, stimulus-responsive regulatory layer in innate and adaptive immunity. Single-cell 3′-end profiling across hematopoietic and lymphoid lineages further demonstrates lineage- and state-specific APA programs, with systematic 3′ UTR shortening of immune effector and signaling genes accompanying activation and differentiation (35, 36).

Consistent with the 3′aQTL analysis, global 3′ UTR changes were observed during the innate immune response to bacterial infections, including *Listeria monocytogenes* and *Salmonella* Typhimurium (35), and during infection of macrophages with vesicular stomatitis virus (37), indicating that infection-driven modulation of 3′-end processing can broadly rewire innate defenses. The immune 3′aQTL atlases show that many APA-sensitive loci encode upstream regulators and negative-feedback components of the NF-κB and MAPK pathways, suggesting that 3′ UTR selection could determine how strongly and for how long these pathways remain active after stimulation. Within this broader landscape, our data place CFIm25 as a key macrophage 3′ UTR-APA regulator whose manipulation is sufficient to override *Salmonella*’s attempt to impose an M2-like, replication-permissive program and instead maintain macrophages in a bactericidal state.

Our current study found that STM reduces CFIm25 levels, and earlier, we showed that CFIm25 is more abundant in M1-polarized macrophages than M2s (14). However, the mechanism responsible for this regulation has not been investigated. Interestingly, we recently reported that the CFIm25 level increased during the differentiation of monocytes to macrophages through a mechanism that does not involve changes in the amount of CFIm25 mRNA but instead uses relief of translational inhibition mediated by PCBP1 (38). A recent study has reported that *Mycobacterium tuberculosis* also targets CFIm25 but through a different mechanism. This pathogen supports its survival in macrophages by disrupting CFIm25’s interaction with another subunit of the CFIm complex (39). In squamous carcinoma cells, the binding of CFIm25 with its partner CFIm68 is strengthened by lactylation (40). Further work is needed to identify the relevant strategies that target CFIm25 levels during *Salmonella* infection.

Changes in subunits of the mRNA 3′-end processing complex or accessory factors are a common mechanism to elicit changes in poly(A) site choice (10–12). Thus, other APA regulators in addition to CFIm25 could be targeted by STM to modulate mRNA fate in a way favorable to bacterial infection. Surprisingly, a 2017 proteomic study of STM-infected HeLa cells reported only 16 significantly downregulated host nuclear proteins, and 7 of these (CstF77, CstF64, CstF50, CPSF160, and CPSF100) were subunits of the polyadenylation complex or other known APA regulators (U1A and CELF1) (41). However, this study did not investigate the relevance of these findings or whether infected macrophages showed similar changes.

Taken together, our findings and the broader APA data sets indicate that 3′ UTR-APA acts as a post-transcriptional “decision layer” that integrates multiple cues to direct macrophage polarization toward either bacterial clearance or tolerance. *Salmonella* remains a prevalent public health problem, which is worsened by increasing antibiotic resistance (42, 43). Our study suggests that strategies to restore or increase CFIm25 activity during infection could represent a host-directed therapeutic approach to enhance macrophage bactericidal function.

## SUPPLEMENTAL MATERIAL

Supplemental material iai.00361-26-s0001.pdf Fig. S1 and S2; Table S1.

ASM does not own the copyrights to Supplemental Material that may be linked to, or accessed through, an article. The authors have granted ASM a non-exclusive, world-wide license to publish the Supplemental Material files. Please contact the corresponding author directly for reuse.
