# Supplementary material for: The alternative polyadenylation factor CFIm25 orchestrates macrophage antibacterial activity against Salmonella through the TAB2 and TBL1XR1 regulators of MAPK and NF-κB signaling
Source: Infect Immun. 2026 Jul 22;94(8):e00361-26. doi: 10.1128/iai.00361-26 (PMC13460137; doi:10.1128/iai.00361-26)
Supplement: Supplementary file 1 [file iai.00361-26-s0001.pdf]

Supplementary Figure 1.

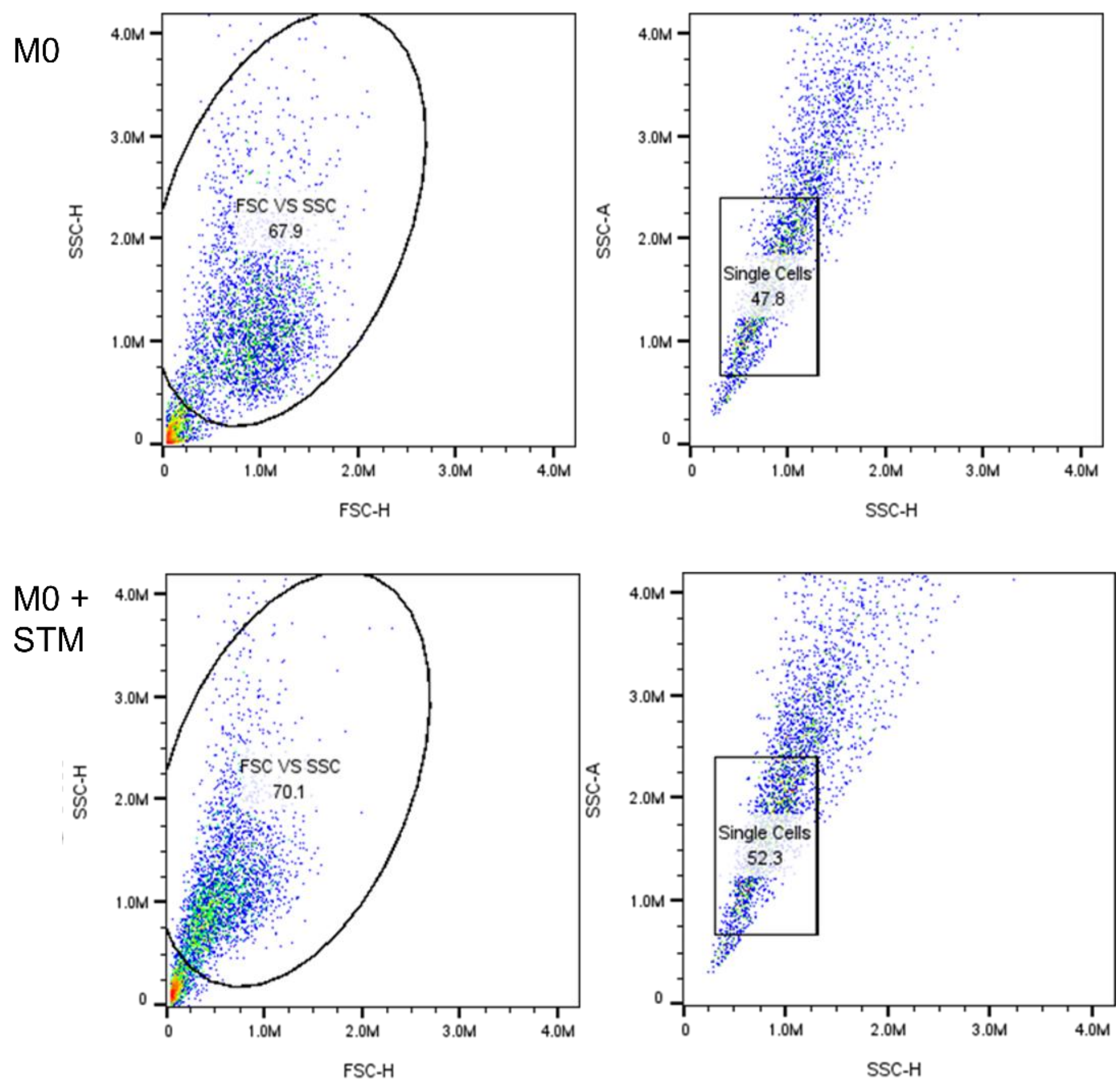

Supplementary Figure 1. Flow cytometry gating strategy for macrophage analysis for M0 and M0+STM samples. Representative gating strategy used for all flow cytometric analyses, where cells were first gated based on forward scatter (FSC) and side scatter (SSC) to exclude debris and define the main cell population. From this population, singlets were identified using SSC-area versus SSC-height plots to exclude doublets and aggregates. Percentages indicate the fraction of events retained at each gating step relative to the parent population. Sequential gating was used to generate a single-cell population for downstream analyses, including surface marker expression, viability, and functional assays.

Supplementary Figure 2

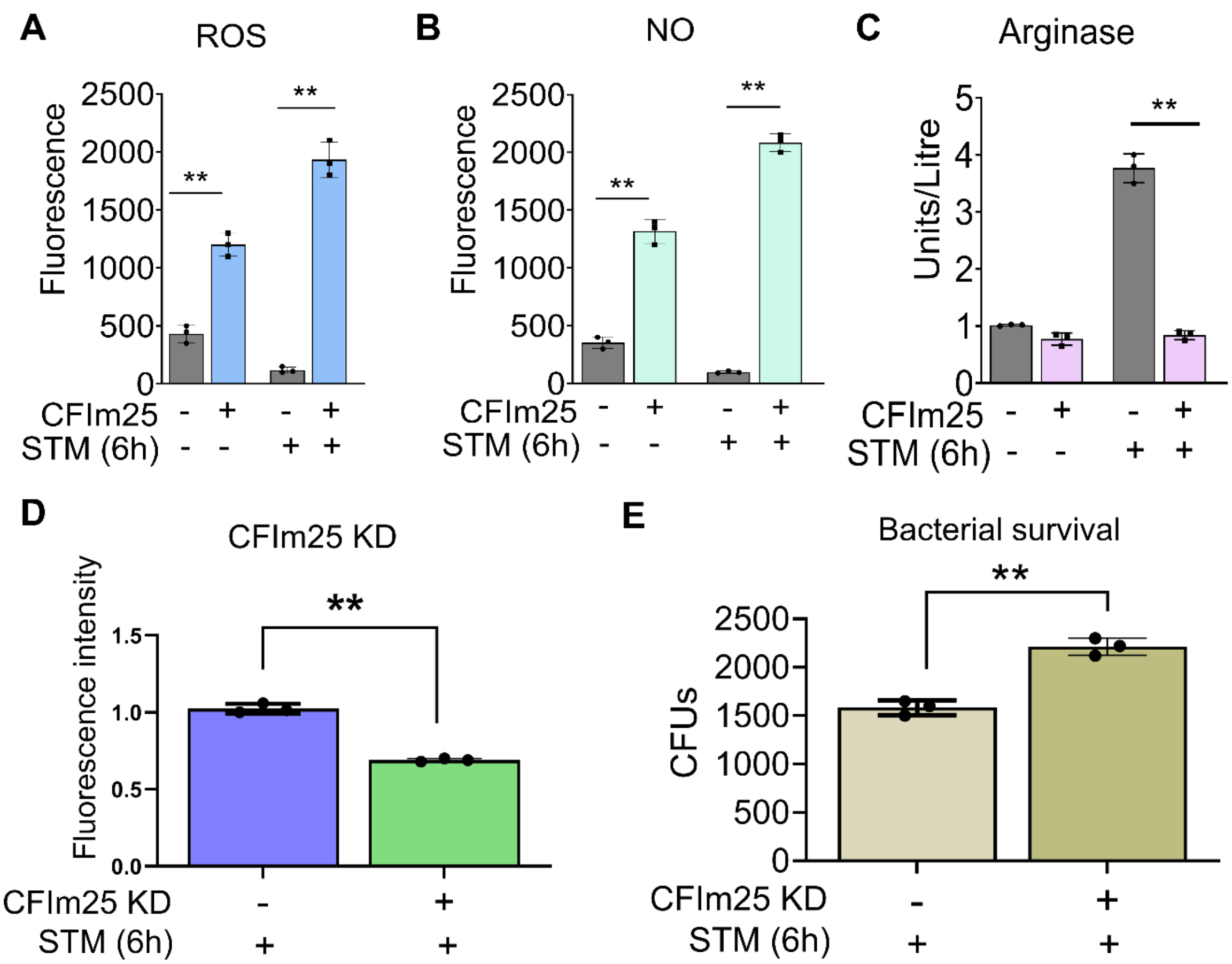

Supplementary Figure 2. CFIm25 overexpression enhances macrophage antimicrobial activity in both uninfected and STM-infected (6 hours) conditions. (A) Flow cytometric quantification of ROS production using the DCFH-DA fluorescence assay in control (-) and CFIm25-OE (+) macrophages under uninfected (-STM) and STM-infected (+STM) conditions. (B) Flow cytometric quantification of NO production using the DAF-FM fluorescence assay in control and CFIm25-OE macrophages under uninfected and STM-infected conditions. (C) Arginase activity measured by urea production in control and CFIm25-OE macrophages under uninfected and STM-infected conditions. (D) Flow cytometric quantitation of CFIm25 levels presented as mean of fluorescence intensity normalized to CFIm25 level in 6-hour STM-infected cells without CFIm25 KD. (E) Intracellular bacterial burden determined by colony-forming units (CFU) in control (-) and CFIm25-KD (+) macrophages infected with STM for 6 hours. Data are from three independent experiments (n = 3); **, P < 0.01.

Supplementary Table 1

TAB2 total	TAB2 long
**Forward**	**Forward**
AGGGAGGGCTGAGGTGTC	AGACCCAAAGCCCTTACGTT
**Reverse**	**Reverse**
CACTAGGGCAGCCGTCTC	ACCCAGCTAAATCACAAAACCA
**TBL1XR1 total**	**TBL1XR1 long**
**Forward**	**Forward**
CTGTCCCTGATAGATGCCGT	TAAACCAGCCCATGACAGGT
**Reverse**	**Reverse**
TTCTCCTCCCCATTTGCTGT	AGGGGAACTGCAACAACAAC

Supplementary Table 1.

Sequences of primers used for RT-qPCR analysis of total and long TAB2 and TBL1XR1 transcripts. Total primers are located in the common coding sequence; long primers are positioned downstream of the proximal poly(A) site to detect transcripts utilizing the distal poly(A) site.
